# Gastric Ulcer

**Published:** 1878-12

**Authors:** E. Dreifus


### GASTRIC ULCER.

By E. DREIFUS, M.D.

This lesion, which, on account of its characteristic form and peculiar course, is designated as *ulcus rotundum or per-*

*forans*, was not known to the older physicians—at least they had no thorough knowledge of it, but confounded it generally with other morbid processes. It was first distinctly described by Cruveilhier, in his great work on pathological anatomy, in the year 1830, he saying it was previously confounded with cancer of the stomach.

In 1839 Rokitansky gave an account of it under the name of perforating ulcer of the stomach. A very fine essay was published by Cruveilhier, in the *Archives Generales* for February and April, 1856. To Dr. William Brinton and his valuable essay are we indebted for many of the facts now known in regard to this disease.

The chief seats of it are at the lesser curvature, posterior wall, and specially in the pyloric portion, and at the cardia. In very rare cases it occurs in the duodenum or œsophagus.

The characteristic features of the ulcer are its circular form, as if stamped out, and its tendency to extend destructively to all the strata of the gastric parietes. The process of destruction always commences at the mucous membrane, and is confined to it in a large number of cases. Accordingly we find not unfrequently in bodies the traces of a previous simple ulcer; and the healing takes place, as in all other ulcerations, by means of the formation of new connective tissue at the bottom of the ulcer, by which the edges gradually grow together and finally unite. In proportion to the loss of substance will be the constriction and shortening, causing deformity of the stomach; and the consequences may be both a narrowing of the pyloric half, and also a considerable interference with the vermicular movements of the organ. But if the ulcer progresses, it then frequently leads to perforation, and, by the escape of the contents of the stomach, gives rise to general and usually fatal peritonitis.

In respect to extent and size, numerous gradations occur, and the form of the stomach is still more irregular when several ulcers become confluent.

*Causes.*—The causes of simple gastric ulcers are not sufficiently known. Probably several factors concur in their production. We may assume, as probable, that a partial disturbance of nutrition, due to disease of the blood-vessels, occasions a circumscribed gangrenous destruction of mucous membrane. The hypothesis that an altered condition of gastric juice gives rise to the ulcer appears to me to be unfounded; nevertheless it cannot be denied that the vermicular movements of the stomach and the action of the gastric juice hinder the cicatrization and consequent healing. Without doubt, similar ulcers occur on other mucous surfaces; but, on the one hand, they are not followed by the same severe consequences as in the simple ulcer of the stomach, and, on the other hand, they heal much more readily. Under unfavorable circumstances, as has been mentioned, the ulcer ends in perforation of the stomach and fatal peritonitis; but this occurrence will rarely be prevented by the circumstance that the base of the ulcer has formed adhesions to some of the neighboring organs. Such adhesions are formed corresponding to the seat of the ulcer, more frequently between the stomach and pancreas or duodenum, and also with the left lobes of the liver, the anterior walls of the abdomen and omentum, the spleen, the diaphragm, the colon, etc. If the loss of substance be small and the adhesions to the neighboring parts firm, life may be prolonged for a considerable period. But if the loss of substance be great, the function of the stomach will, in spite of the cicatrization, be much disordered, and the nutrition of the animal economy will suffer severely in consequence. Besides, even with firm adhesions, subsequent perforations may occur from softening of the false membranes.

*Symptoms.*—The symptoms which accompany ulcer of the stomach during life are very variable. Sometimes for a long interval the symptoms are very insignificant, or may be entirely absent; but, for the most part, disorders of the stomach manifest themselves. Generally we observe a very painful sensation in the epigastrium, of weight, or drawing together. By pressure in the region of the stomach, a fixed, painful spot is detected. But these phenomena are also manifested in chronic gastric catarrh, and in carcinoma of the stomach; and either of these complaints may be confounded with simple gastric ulcer.

The appetite is usually more or less disturbed, occasionally unchanged and ofttimes increased. Yet the patients complain of slow digestion after meals, of pains, of pyrosis, eructations, etc. As the disorder increases, retching and vomiting make their appearance. The pain is generally fixed, but not confined to the same spot. All these symptoms, as is evident, are not pathognomonic, and physicians are, therefore, at an early period of the disease, not in a position to make a positive diagnosis. The hæmatemesis is of greater importance, and it is also one of the most dangerous symptoms, from its dreaded tendency to collapse. Vomiting of blood occurs with varied intensity. The vomited matters are either only slightly tinged with blood, or are colored chocolate brown, or like coffee grounds, the dark color arising from the action of the gastric juice upon the blood effused into and detained for some time in the stomach.

Should, during the course of ulceration, a larger blood vessel be eroded, the hemorrhage might be sufficient to cause immediate death, or at all events the highest degree of anæmia, and exhaustion would result. A feeling of weight and fullness of the epigastrium frequently precedes the vomiting of blood. The hæmatemesis may take place at any period of the disease. The results of profuse vomiting of blood are similar to hemorrhages all over the body—syncope, pallor, coldness of the extremities, feeble pulse, etc. Sometimes hemorrhage takes place without vomiting. If a patient suddenly turns pale after a momentary feeling of weight and heat in the epigastrium, and, on examination, the region of the stomach yields a hollow percussion sound; if the pulse becomes feeble, and syncope comes on, from these symptoms we may conclude that internal hemorrhage has taken place. Such an internal hemorrhage may occasion death without vomiting, as the bleeding generally occurs during digestion. Bodily and mental emotions may induce it, but especially any excitement of the circulation. Emetics, also, for which the patient often craves, may bring it on.

Several stages of this disease may be distinguished. In the first, the formation of the ulcer occupies a considerable time for its completion, the chief symptom being simply a kind of gastralgia, sometimes, indeed, of a most intense degree. The pains present nothing characteristic; they may be continuous and fixed or paroxysmal, and may be very easily mistaken for nervous gastralgia. The occurrence of pain in the spine opposite the epigastrium is also not characteristic, being found in other gastric affections. Hence, in the early stage, ulcer of the stomach is very difficult to diagnose. Palpation reveals at most a fixed spot, where pain is increased by pressure, and only in the case of persistent adhesions can we sometimes discover a certain induration.

In the succeeding stage, vomiting of blood comes on, from which we are better able to decide on the nature of the disease, although this symptom does not exclusively belong to simple ulcer of the stomach, but does sometimes appear in the course of carcinoma of that organ. Even in this stage of the disease, Drs. Brinton and Budd say: "Often repeated hemorrhages have taken place; the process of healing by cicatrization does sometimes occur, and patients do get well." It has been my lot to see only two cases, and both proved fatal. Hemorrhage must always be regarded as a very grave symptom, because the bleeding itself may prove dangerous. And, besides, it indicates that deeper ulceration is in course of progress. Usually all the blood is not vomited, but a portion passes off by the stools, in an altered condition, and sometimes the whole of the effused blood is so discharged.

In the third stage, perforation of the mucous membrane takes place, in consequence of which the contents of the stomach escape into the cavity of the peritoneum, causing a usually rapid and fatal peritonitis. This can only be averted, in the case of slowly formed perforation, by adhesions to the neighboring parts, and sometimes these adhesions give way at a later period. If these adhesions are extensive, and give rise to a hardness perceptible to the touch, they may be confounded with carcinoma. Occasionally perforations occur suddenly, not preceded by other considerable symptoms of disease, as for instance when the progress of the ulcer is quite latent. Extensive adhesions may occasion long continued disorders of the stomach and induce ill health ; but a small adhesion may remain after cure without producing any derangement of the stomach whatsoever.

The morbid appearances to be looked for after death are a smooth, round, ulcerated spot, as if stamped out, and adhesions. We know that gastric *post-mortem* changes occur early, and are sometimes due to cadaveric digestion, as well as hypostases and putrefaction ; and they have sometimes been misinterpreted as the *ante-mortem* lesions of inflammation, ulceration and perforation. There are few dead bodies in which the stomach is not in some degree digested. Its greatest ravages are found in the bodies of those suddenly killed after a hearty meal, especially if the body has been kept in a warm place. In such cases the stomach may be perforted with ragged, lacerated openings, and its contents be found floating in the abdominal cavity; or even greater ravages may ensue.

Cadaveric digestion sometimes presents erosions enough to simulate ulceration; and drops of blood may flow from the digested ends of small vessels, when pressure is made on the branches from which they are derived.

From the above facts it is manifest that, since engorgement with discoloration, softening, opening of the vessels and destruction of tissues do occur in the most depending part of the stomach, as results of hypostatic, digestive or putrefactive *post-mortem* changes, too great caution cannot be exercised in attributing any such changes to *ante-mortem* lesions, when these changes are limited to its splenic end and to the line of gastric contents.

*Prognosis.*—the prognosis of ulcer of the stomach is always doubtful, although many cases of cure are said to have occurred, and although authorities say a cure may take place at any stage of the disease, I shall always consider it a very grave and serious, if not fatal malady. Death results either from hemorrhage or peritonitis; or, when the disease is of long duration, from exhaustion. From various statistics I have found that nearly one-third of all known cases of simple ulcer of the stomach prove fatal.

*Treatment.*—As regards treatment, little is to be said beyond hygienic measures and nourishment, as there are no specifics for this complaint. The most important rule is, that the patient subject himself to a most rigid dietetic regimen and observe the strictest quietude, in order, if possible, to favor the cicatrization of the ulcer. Besides this, we must endeavor to combat particular distressing symptoms. Milk diet is certainly the best that can be used; and, in consequence of the great irritability of the stomach and the difficulty of patients' retaining any food, I would suggest feeding by the rectum, as we now know that absorption goes on just as readily there as *per viam naturalem;* and we consequently lessen thc peristaltic action of the stomach, which seems to be one of the prime causes that interfere with cicatrization. To allay the gastralgia, hypodermic injections of one of the salts of morphia and other narcotics may be used, and for the frequent constipation, enemata may be employed; for the obstinate vomiting, ice, alum or tannin, and small quantities of carbonic acid waters; for hæmatemesis, ice, alum, tannin, bismuth, hypodermic injections of fluid extract of ergot or ergotine, besides the tincture ferri chloridi; and, in addition, what appears to me the most rational of all, is thc frequent washing out of the stomach with a stomach-pump, using a three per cent. solution of carbolized water.

The greatest obstacle towards a successful treatment seems to be that the chief indications, absolute rest and abstinence from everything injurious, cannot be fulfilled. We are therefore compelled to confine our efforts to reducing the action of the stomach to its minimum, using the most easily digested food, and to feed *per rectum.* This course of treatment must be persevered in for a long time, alternating when the rectum becomes irritable, as it will usually do, and feeding by the mouth again, but only in the most minute quantities, and such articles as require little or no digestion. For perforation little, of course, can be done, and treatment of symptoms is alone available. *New Orleans Med. and Surg. Journal.*

